# Lateralization Technique and Inferior Alveolar Nerve Transposition

**DOI:** 10.1155/2016/4802637

**Published:** 2016-06-28

**Authors:** Angélica Castro Pimentel, Marco Antonio Sanches, Gabriel Cardoso Ramalho, Caio Vinicius Roman-Torres, Marcello Roberto Manzi, Wilson Roberto Sendyk

**Affiliations:** ^1^Department of Post Graduation, Division of Implantology, School of Dentistry, University of Santo Amaro (UNISA), Sao Paulo, SP, Brazil; ^2^School of Dentistry, University of Santo Amaro (UNISA), Sao Paulo, SP, Brazil; ^3^School of Dentistry, Department of Oral and Maxillofacial Surgery and Traumatology, University of São Paulo (USP), Sao Paulo, SP, Brazil; ^4^Graduate Program in Implant Dentistry at the University of Santo Amaro (UNISA), Sao Paulo, SP, Brazil

## Abstract

Bone resorption of the posterior mandible can result in diminished bone edge and, therefore, the installation of implants in these regions becomes a challenge, especially in the presence of the mandibular canal and its contents, the inferior alveolar nerve. Several treatment alternatives are suggested: the use of short implants, guided bone regeneration, appositional bone grafting, distraction osteogenesis, inclined implants tangential to the mandibular canal, and the lateralization of the inferior alveolar nerve. The aim was to elucidate the success rate of implants in the lateralization technique and in inferior alveolar nerve transposition and to determine the most effective sensory test. We conclude that the success rate is linked to the possibility of installing implants with long bicortical anchor which favors primary stability and biomechanics.

## 1. Introduction

With the loss of teeth, the alveolar ridge undergoes a continuous and irreversible process of bone resorption in height and thickness. Thus, mainly the posterior bone resorption sextant jaw usually leads to a reduced bead, and therefore the installation of implants in these regions becomes a challenge [1, 2].

Initially, the surgical protocol proposed by Branemark to treat edentulous mandible patients was the installation of implants in the anterior mandible between the mental foramen [3]. The clinical protocol for rehabilitating subsequent partial edentulous patients preserving the healthy anterior teeth and preventing dental euthanasia, which is currently not a solution adopted by the majority of patients and professionals, was modified and initiated the installation of implants in posterior regions of the jaw [4].

Several surgical techniques have been developed for the rehabilitation of atrophic jaws with the installation of dental implants [5], such as the use of devices for distraction osteogenesis [6], bone grafts [7], guided bone regeneration [8], short implants [9], implant installation sideways to the nerve [10], and the lateralization of the inferior alveolar nerve (LIAN) [1, 11, 12]. For the revising of the atrophic posterior mandible, there are two techniques regarding the alveolar nerve, the first of which calls for the inferior alveolar nerve transposition (IANT) through a bone window created in the cortical bone of the jaw, posterior to the mental foramen without including it. The second method includes the mental foramen in the bone window created and is called the inferior alveolar nerve transposition (IANT) [13, 14]. Smiler [15] notes that the nerve mobilization with the involvement of the mental foramen allows the placement of implants in the region of the canines and the bicuspids. An osteotomy may be performed with rotary tools, drills, or reciprocal devices such as piezoelectric ultrasound technology [16].

The lateralization technique for the inferior alveolar nerve (LIAN) allows for the installation of implants to correct the positioning or to move them closer to the ideal, improving the possibility of direct view at the time of surgery [17]. Using the higher cortical and basal body of the mandible, the implant is encased in a better-quality bone, unlike the reconstruction implants installed in the region with grafts [18]. Compared to the reconstruction methods with grafts, the lateralization procedure does not require donor areas, which decreases patient morbidity, lowers costs, provides ready installation of long implants (because it uses all the remaining jaw bone), and prevents patients from waiting six to eight months for treatment [1].

The posterior mandible has a higher quantity of narrow bone when compared to chin symphysis region that has more cortical bone. The LIAN technique provides a biomechanically favorable result to chewing loads occurring in the posterior region of the mandible. This technique establishes a good proportion between the implant length and the prosthesis length [19] compared to the use of short implants to preserve the mandibular canal, which presents lower initial stability and poor biomechanics that have been impaired by having a unicortical anchor [20].

Jensen and Nock [19] were the first to report the rehabilitation of the atrophic posterior mandible using dental implants in conjunction with inferior alveolar nerve transposition. The technique was a modification of the method used by Alling [21], who performed lateralization surgery on the inferior alveolar nerve to slow the uncomfortable situation of denture patients with an extremely resorbed mandible, where the pressure exerted by the prosthesis on the neurovascular bundle caused pain, making it difficult to feed the patients.

Dao and Mellor [22] observed that, in LIAN procedures, all patients had transient sensitivity disorders of the inferior alveolar nerve, and they reported that this high-risk treatment option should not be considered as a routine solution.

## 2. Objectives

The objective of the case report was to present the lateralization technique and the transposition of inferior alveolar nerve and to evaluate the following: the success rate of dental implants and the most effective sensory test.

## 3. Case Presentation

### 3.1. Patient 1

A female patient, 40 years old, was referred for dental care in the specialty area of implantology with dental absence in the posterior region of the bilateral jaw. After completing the examination and oral clinical assessment, the patient underwent laboratory tests for preoperative evaluation (complete blood count, fasting blood glucose, coagulation, calcium and phosphorus levels, and alkaline phosphatase). After planning, we opted for treatment with dental implants with the LIAN technique in the left mandibular region and IANT in the right mandibular region. Approval for the treatment was documented after clarification of the risk of temporary or permanent paresthesia related to LIAN and IANT and of the risks related to the possible failure of the implant treatment.

The patient used the following as preoperative and postoperative medication starting 24 hours before surgery: Amoxicillin (500 mg Amoxicillin® Medley), 1 tablet every 8 hours for 7 days; Dexamethasone (Decadron® 4 mg AChE), 1 tablet 1 hour before surgery, as a sedative; and 1 tablet of midazolam maleate (7.5 mg Midazolam Hydrochloride® Roche). The surgery was started in an outpatient setting, and the patient's blood pressure was monitored.

#### 3.1.1. Transposition Technique for the Alveolar Nerve, Right Side

*Anesthesia.* The solution used was anesthetic mepivacaine hydrochloride + epinephrine (Mepiadre 100® DFL), 1 plastic tube for the truncal block of the IAN, and 1/2 cartridge infiltrating the area of the mental foramen; 2 tubes hydrochloride Articaine + epinephrine (Articaine 100® DFL) infiltrated the buccal and lingual areas.

**Figure 1:**
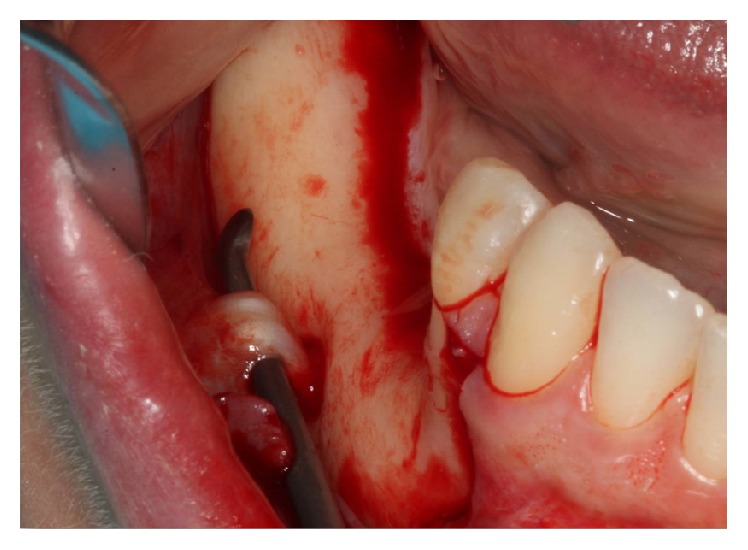
Incision and divulsion.

*Incision and Divulsion.* A crestal incision (15C scalpel blade) was performed in the retromolar trigone region to the neck of tooth 44, followed by an intrasulcular incision on the same tooth and a horizontal incision between teeth 44 and 43, maintaining their papilla, and ending with a relaxing incision in the distal tooth 43 and the distal end of crestal incisions buccally. The flap was carefully and completely removed in the right mental foramen region where the periosteum is performed in the lower region of the mental foramen, extending to the base of the jaw (Figure 1). 

**Figure 2:**
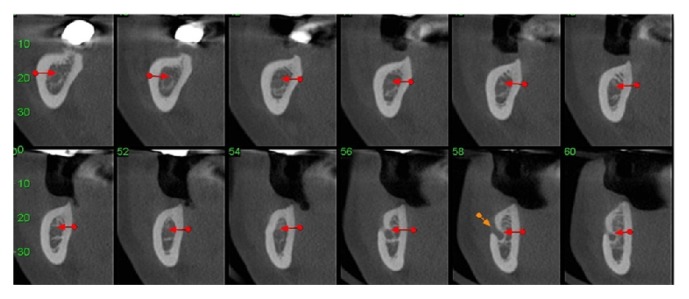
Computed tomography (cone beam) of the jaw, right side.

**Figure 3:**
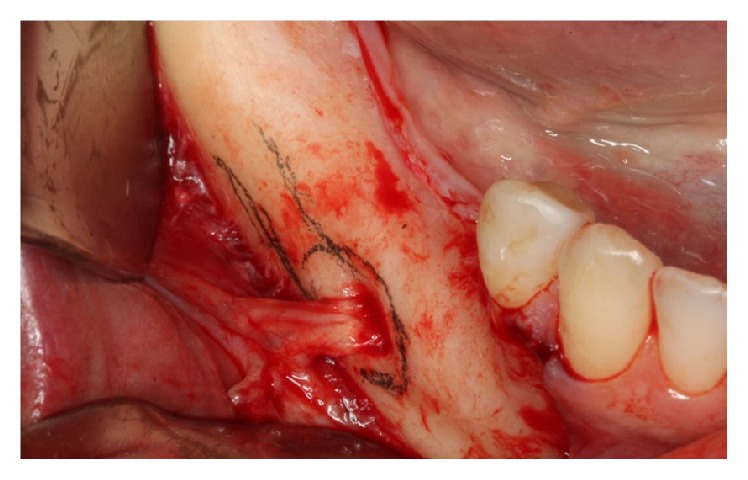
Definition of the mental foramen and the mandibular canal.

**Figure 4:**
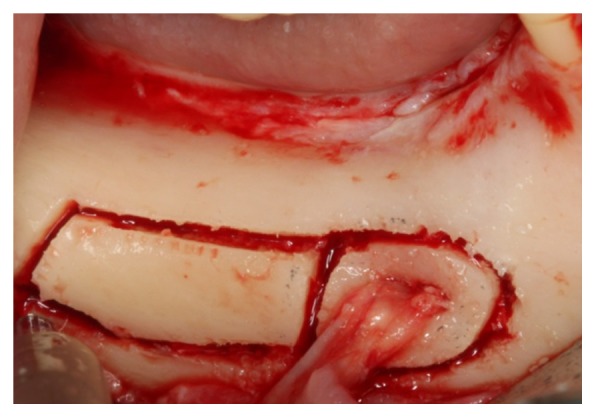
Osteotomy lateral to the mandibular canal involving the mental foramen.

*Osteotomy.* Using CT (cone beam), the molar regions of the right jaw were observed and cutting lines for the osteotomy were planned for the remaining bone volume with 5.4 mm thickness and 4.8 mm height (Figure 2). Using a ruler, needle point, and a pencil of sterilizable graphite, it was possible to plan and carry it to the surgical area, thus delimiting the mental foramen and the mandibular canal, the vestibular bone surface of the body jaw, always with a 2.0 mm safety margin for all traits (Figure 3). The lateral osteotomy to the mandibular canal was performed on the lines of the piezoelectric ultrasound using the tip OT 7 for bone cutting, involving the mental foramen, extending to the cancellous bone (Figure 4). During osteotomy, irrigation was performed with sterile distilled water.

**Figure 5:**
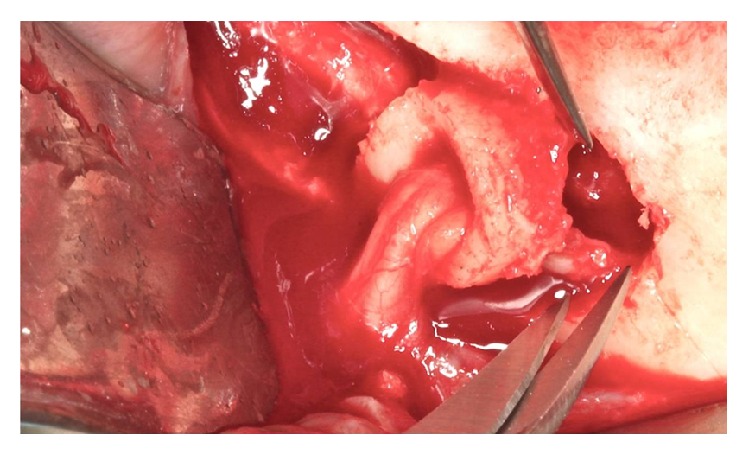
Section of the incisive nerve.

**Figure 6:**
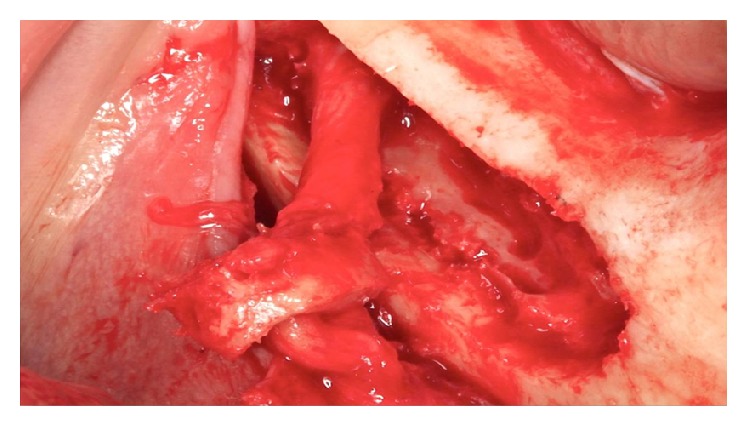
Mental foramen displacement.

*Installation of Implants.* After the osteotomy, the cleavage of the buccal bone plate was performed with a Freer elevator, keeping the mental foramen in position. Then, the displacement of the mental foramen was performed with the Freer, and with Goldman Fox scissors the incisive nerve was severed (Figure 5). The cortical bone in the chin region emerging nerve was removed, and the displacement of the mental foramen was carried distally (Figure 6).

**Figure 7:**
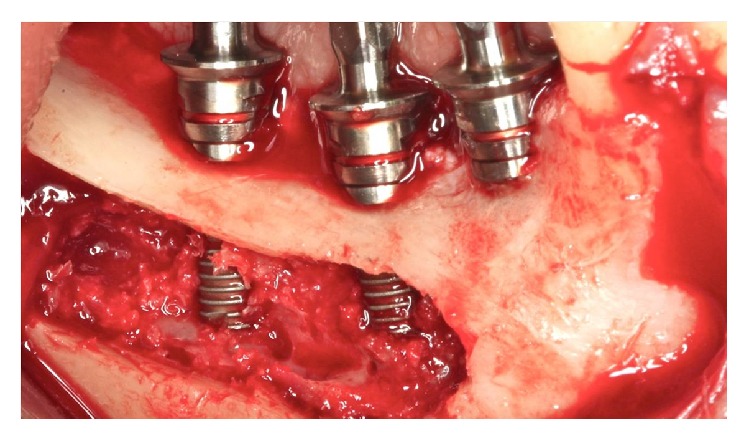
Installation of implants.

The milling and installation of implants with locking bicortical were implemented in the regions of tooth 45 (LTX XP® 3.25/4.1 × 13 mm, Biomet 3i), tooth 46 (LTX XP 3.25/4.1 × 13 mm Biomet 3i), and tooth 47 (LTX XP 3.25/4.1 × 11.5 mm, Biomet 3i) (Figure 7). 

**Figure 8:**
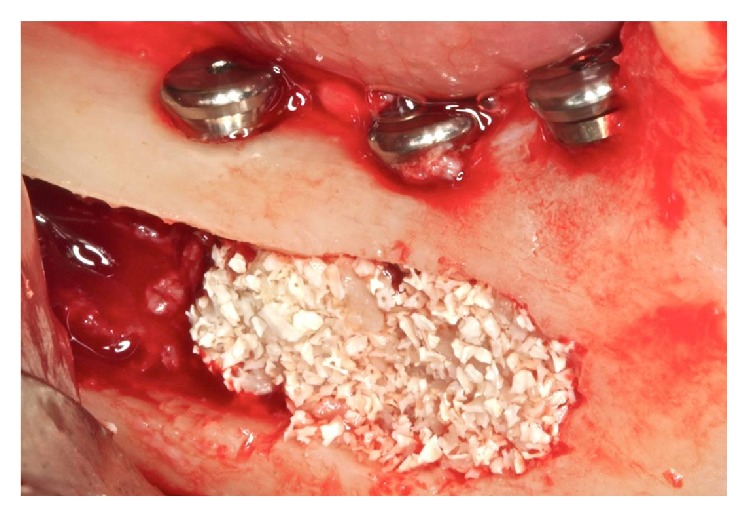
Filling bone.

**Figure 9:**
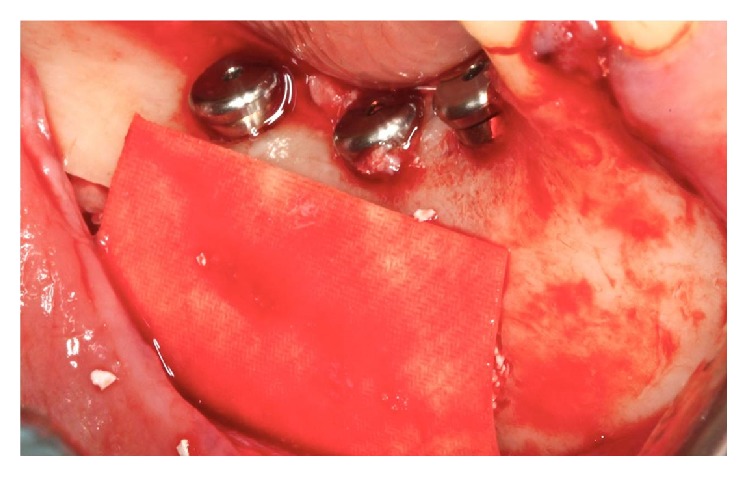
Placement of absorbable membrane.

**Figure 10:**
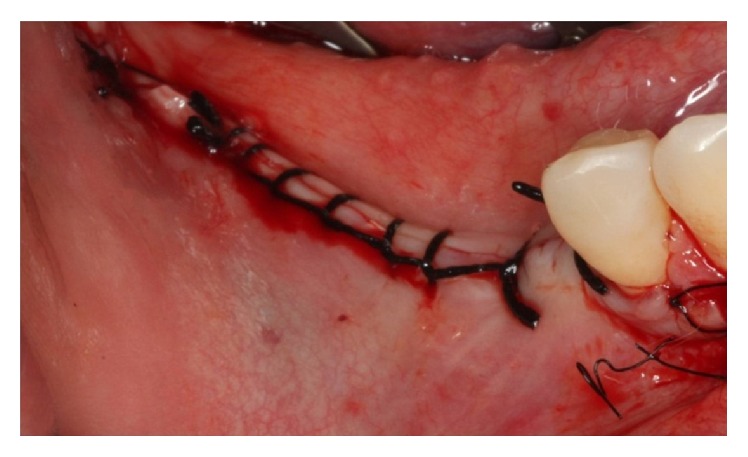
Suture.

*Bone Grafting and Suture.* The vestibular bone block was removed particles for coating the turns and fill the window. Only placed autogenous bone was in contact with the exposed turns in the middle portion of the implants. On this layer was placed autogenous bone combined with bovine hydroxyapatite (Endobon®, Biomet 3i, USA). At this time, the mental foramen rested in the distal region of the posterior implant, and thus the IAN was seated on this bed (Figure 8). The protection of the graft was performed with an absorbable membrane (OsseoGuard*™*, Biomet 3i, USA) (Figure 9). After that, the flap was sutured using 3-0 silk thread (Ethicon®) and 5-0 nylon thread (Ethicon) in the relaxing incisions (Figure 10).

#### 3.1.2. Lateralization Technique for Alveolar Nerve, Left Side

The steps of anesthesia, incision, and dilatation were similar to those described above regarding the surgery on the right side.

**Figure 11:**
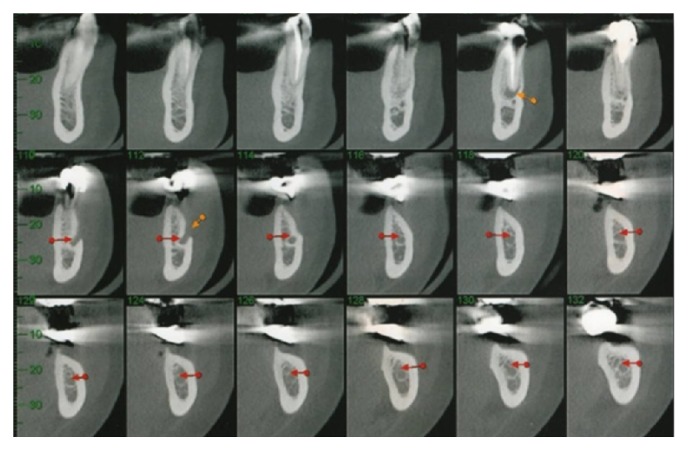
Computerized tomography (cone beam) left jaw.

We used computerized tomography to observe regions of the molar with volume 5.0 mm, average height, and 8.0 mm thickness. In tooth 35, there was a sufficient quantity of bone for implant installation, making it unnecessary to involve the mental foramen (Figure 11). 

**Figure 12:**
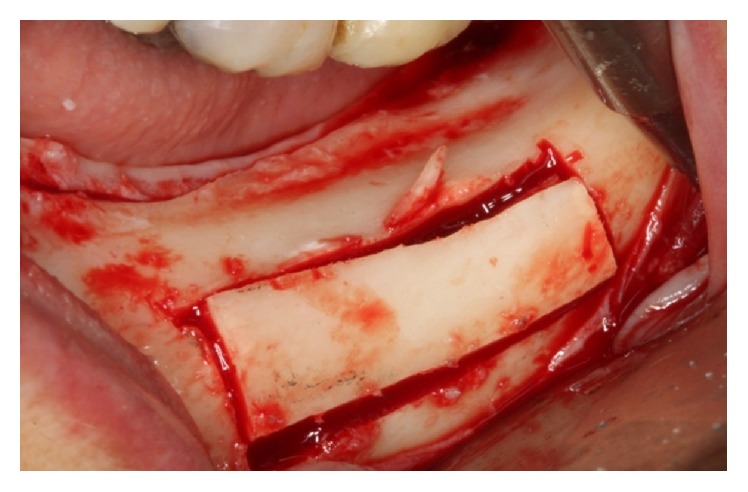
Osteotomy.

*Osteotomy.* The line of the upper horizontal osteotomy was performed 2.0 mm above the upper cortical bone of the mandibular canal; it was initiated 2.0 mm distal to the mental foramen and extended about 7.0 mm posterior to the distal implant, with the goal of not distending the neurovascular bundle too much. The lower horizontal osteotomy was performed 2.0 mm below the lower cortical bone of the mandibular canal. Two vertical osteotomies connected the two horizontal cuts, which were made 2.0 mm distal to the mental foramen as a safety margin (Figure 12). 

**Figure 13:**
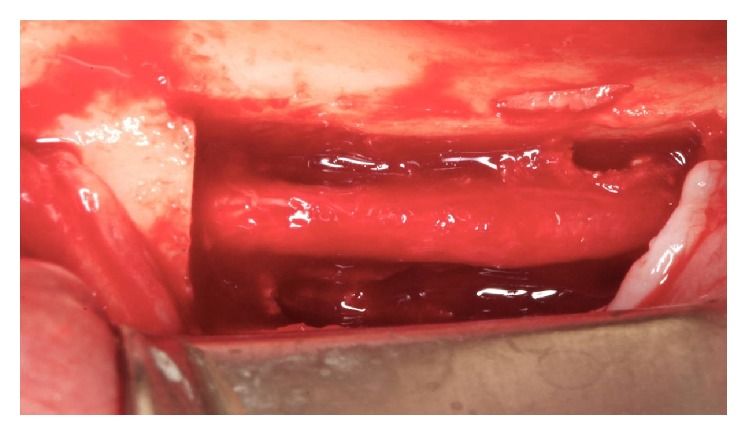
The inferior alveolar nerve's appearance after removal of bone tissue.

**Figure 14:**
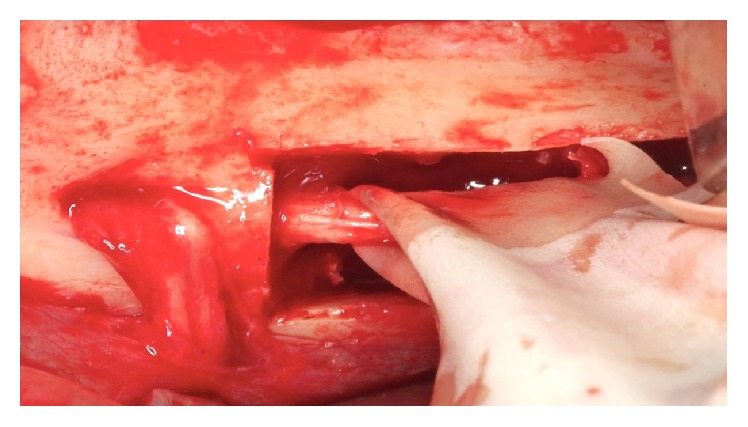
Traction beam with latex.

**Figure 15:**
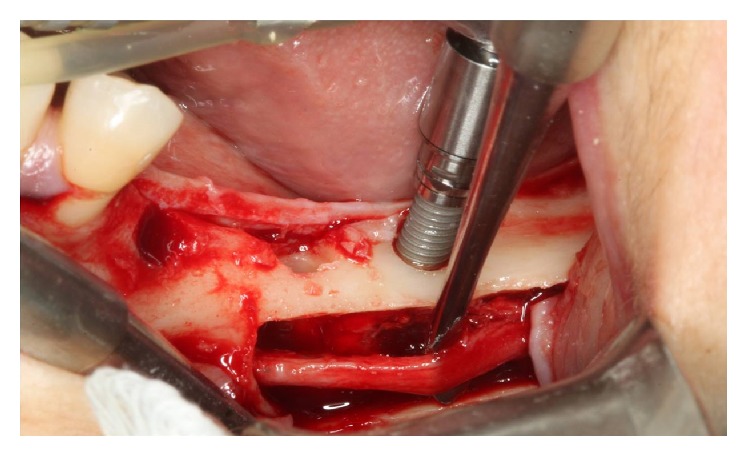
Traction beam performed with a Freer.

*Handling the Lower Alveolar Nerve.* The cleavage of the cortical bone was performed with a Freer. With the aid of a spherical diamond tip on the piezoelectric device, the cortical bone was removed throughout the cancellous bone around the neurovascular bundle (Figure 13). With a Freer and a sterile glove fragment, the beam was pulled buccally to the milling procedure and the implant placement (Figures 14 and 15).

**Figure 16:**
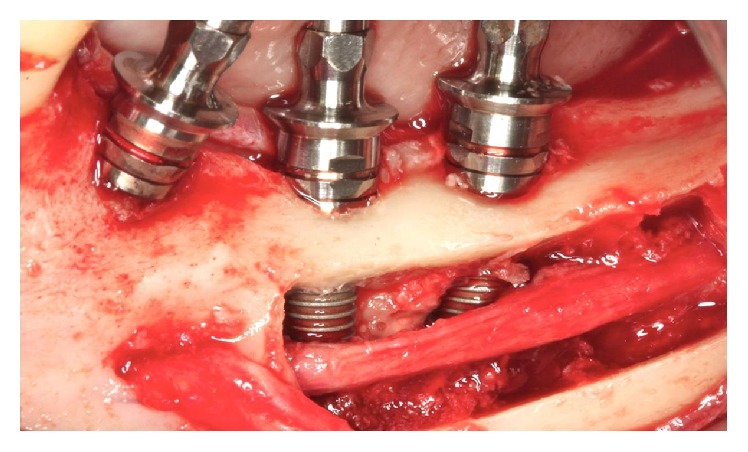
Implants were installed.

The implants were installed in the regions of the teeth 36 (LTX 3.5/4.1 × 13 mm, Biomet 3i) and 37 (LTX XP 3.25/4.1 × 11.5 mm, Biomet 3i). In the region of the tooth 35, the implant has been positioned by conventional milling technique (XP LTX 3.25/4.1 × 8.5 mm, Biomet 3i) (Figure 16).

**Figure 17:**
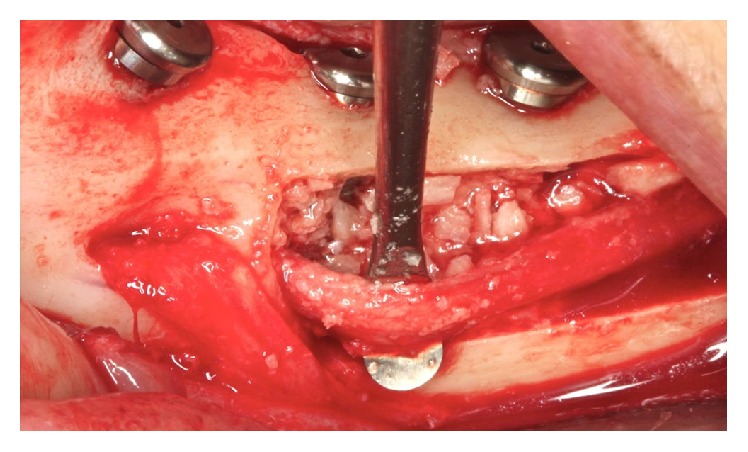
Filling in contact with implants (autogenous bone).

**Figure 18:**
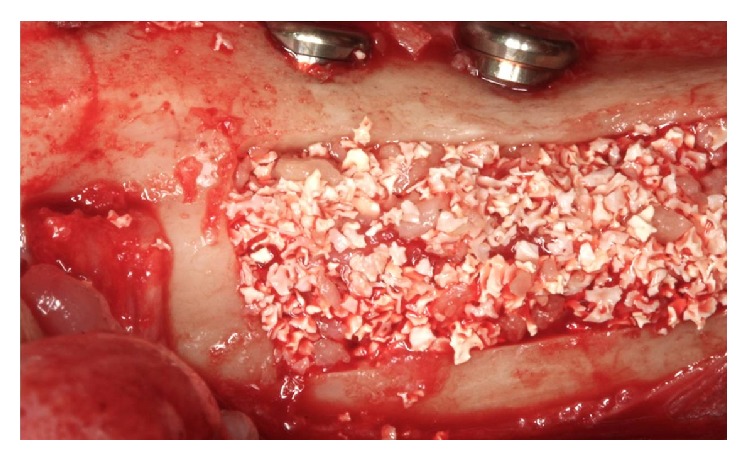
Intermediate fill (autogenous and hydroxyapatite bone).

The vestibular bone block was removed and particles were used to coat the turns and fill the window. Autogenous bone was put in contact with the exposed turns in the middle portion of the implants. Autogenous bone hydroxyapatite combined with bovine hydroxyapatite (Endobon, Biomet 3i, USA) was placed in an intermediate layer (Figure 17). At this point, the IAN was repositioned over the graft and a new layer of hydroxyapatite with autogenous bone was placed completely involving the nerve bundle (Figure 18). Filling out the window is finished using only bovine hydroxyapatite (Endobon, Biomet 3i, USA).

**Figure 19:**
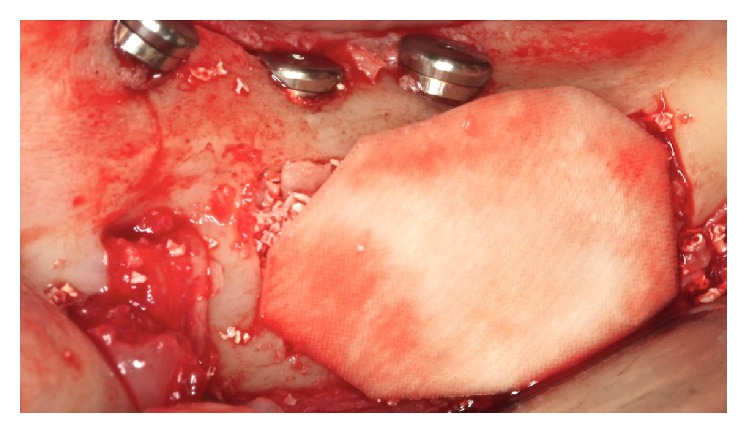
Placing the absorbable membrane.

**Figure 20:**
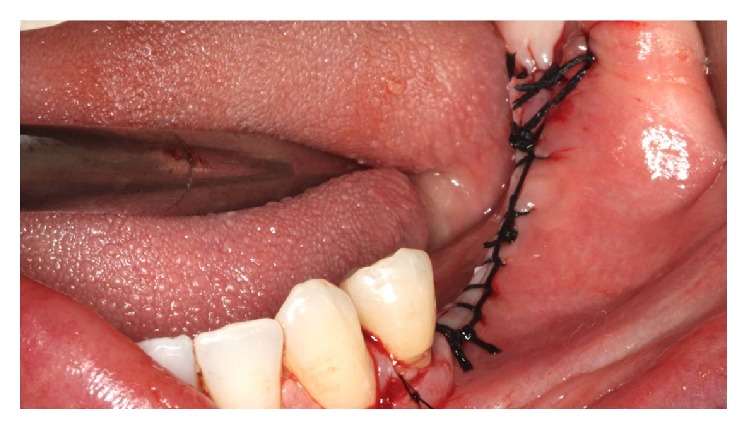
Suture.

The absorbable membrane (OsseoGuard Biomet 3i, USA) was inserted on the grafted area (Figure 19). The flap was repositioned with a scalloped continuous suture with 3-0 silk thread (Ethicon) and 5-0 nylon thread (Ethicon) in the relaxing incisions (Figure 20). 

**Figure 21:**
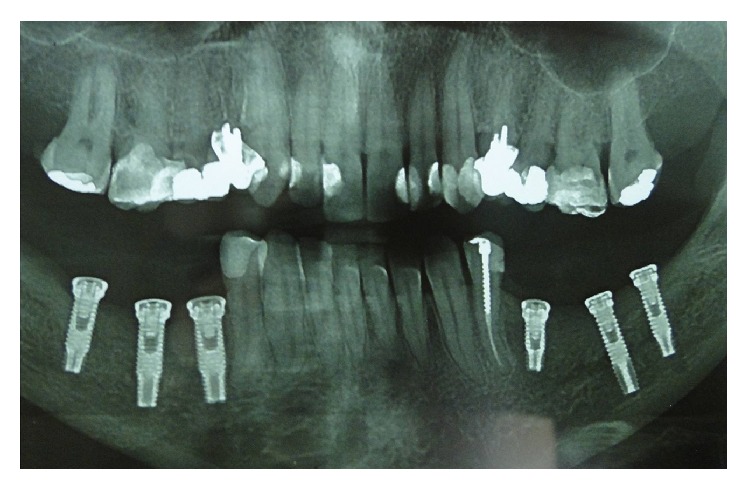
Panoramic X-rays.

*Postoperative Recovery.* The patient was advised to use anti-inflammatory nimesulide (nimesulide, Medley, 100 mg), 1 tablet every 12 hours for 5 days, and analgesic dipyrone (sodium dipyrone, Medley, 500 mg), 30 drops every 6 hours for 2 days. After surgery, a panoramic radiography was performed to evaluate the implants (Figure 21).

The postoperative signs and symptoms were swelling, bruising, and loss of feeling in the region but the right side was more evident than the left side.

The patient underwent low-power laser applications (laser Thera, DMC, São Carlos, Brazil) every 3 days for 4 weeks. The sessions were held with the laser using low infrared power (840 nm and 120 mw) continuously and in a timely manner (1 point per cm^2^) for 30 seconds, both intra- and extraorally, following the inferior alveolar nerve path on the right and left sides.

The patient underwent disodium phosphate cytidine (uridine-5′-triphosphate trisodium) hydroxocobalamin acetate (Etna®, Gross), 4 tablets a day (2 tablets after each meal) for 30 days. Weekly mechanical tests were carried out with the intention of observing the restoration of sensitivity in surgical sites. After 30 days, the patient reported significant improvement in sensory changes; a reduction in both tingling and anesthesia was reported. At this time, they stopped using the drug and ceased the laser therapy sessions. The total return of sensorineural activity occurred in three months.

In conclusion, the postoperative complaint was loss of feeling in the region. 

**Figure 22:**
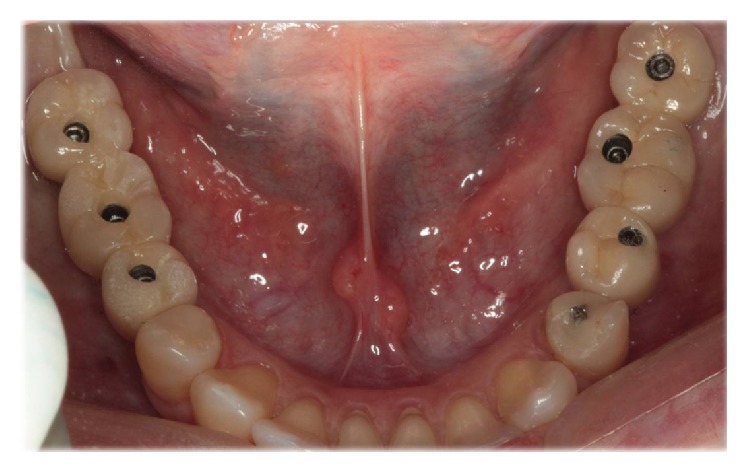
Clinical aspect of prosthetic rehabilitation.

**Figure 23:**
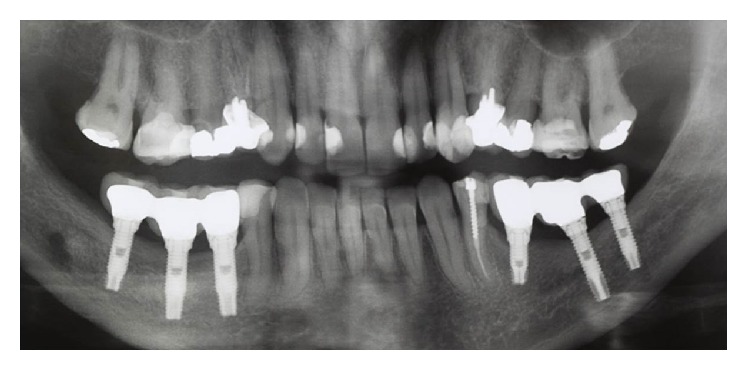
Panoramic X-rays after prosthetic rehabilitation.

*Prosthetic Phase.* The prosthetic stage started after six months. Initially, fixed prostheses were made in indirect bolted fixed resin prostheses were created. After three months, PFM ferulized prostheses were made (Figures 22 and 23).

### 3.2. Patient 2

A male patient, 55 years old, was referred for dental care in the specialty of implantology with dental absence in the posterior region of the bilateral jaw. After preoperative and planning procedures, we opted for treatment with osseointegrated implants and LIAN in the right mandibular region. Approval for treatment was documented after clarification of the risks of temporary or permanent paresthesia related to the IAN and the risks related to the possible failure of the implant treatment.

The patient used the following as preoperative and postoperative medication starting 24 hours before surgery: Amoxicillin (500 mg Amoxicillin Medley), 1 tablet every 8 hours for 7 days; Dexamethasone (Decadron 4 mg AChE) 1 tablet 1 hour before surgery, as a sedative; and 1 tablet of midazolam maleate (7.5 mg Midazolam Hydrochloride Roche). The surgery was started in an outpatient setting, and the patient's blood pressure was monitored.

#### 3.2.1. Anesthesia

The anesthetic used was the solution hydrochloride mepivacaine + epinephrine (Mepiadre 100 DFL), 1 plastic tube for the truncal block of the IAN and 1/2 cartridge infiltrating the area of the mental foramen; 2 tubes of Articaine + epinephrine hydrochloride (Articaine 100 DFL) infiltrated the buccal and lingual areas.

#### 3.2.2. Incision and Divulsion

**Figure 24:**
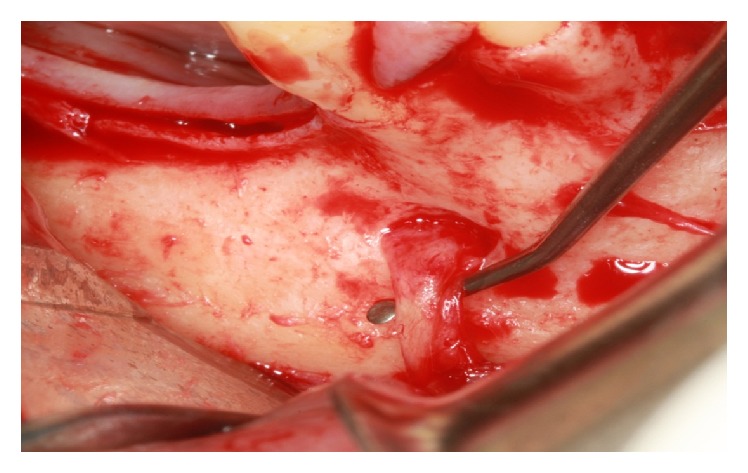
Mental isolated nerve.

A crestal incision (15C scalpel blade) was performed in the retromolar trigone region of the neck of tooth 44, followed by an intrasulcular incision on the same tooth and a horizontal incision between teeth 44 and 43, maintaining their papilla, and ending with a relaxing incision in the distal tooth 43 and the distal end of crestal incisions buccally. The flap was carefully and completely removed. The chin nerve was isolated (Figure 24).

#### 3.2.3. Osteotomy

**Figure 25:**
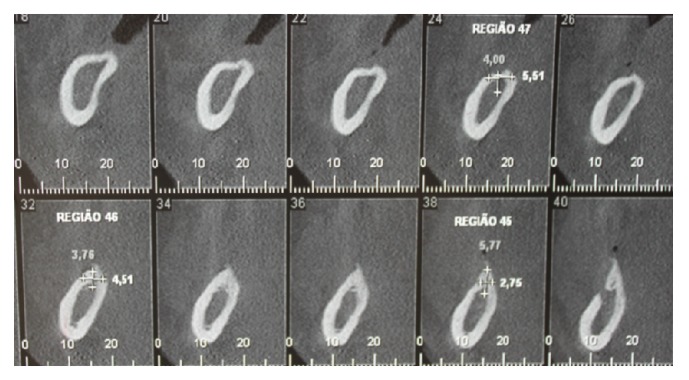
Computed tomography (cone beam) of the right mandible.

Using CT (cone beam), on the right side of the mandible, the remaining bone volume that was 4.51 mm high and 3.75 mm wide was observed in the region of tooth 45; in the region of tooth 46, it was 5.77 mm high and 2.75 mm wide; and in tooth 47, it was 4.0 mm high and 5.51 mm wide (Figure 25).

**Figure 26:**
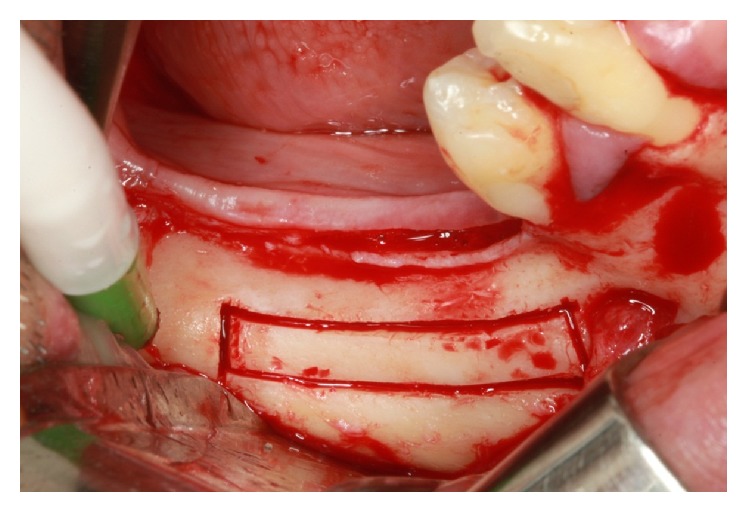
Osteotomy.

The line of the upper horizontal osteotomy was performed 2.0 mm above the upper cortical of the mandibular canal, initiated 2.0 mm distal to the mental foramen and extending about 7.0 mm posterior to the distal implant, with the goal of not distending the neurovascular bundle too much. The lower horizontal osteotomy was performed 2.0 mm below the lower cortical of the mandibular canal. Two vertical osteotomies connected the two horizontal cuts, which were made 2.0 mm distal to the mental foramen (Figure 26).

#### 3.2.4. Handling the Lower Alveolar Nerve

**Figure 27:**
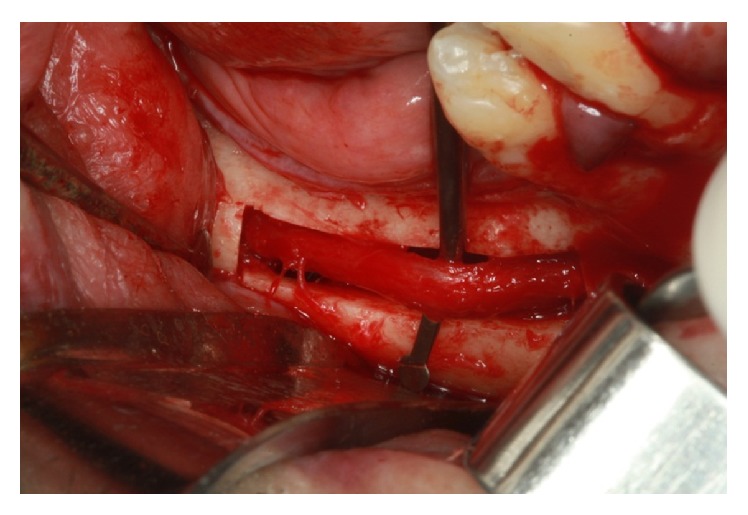
Traction beam performed with a Freer.

**Figure 28:**
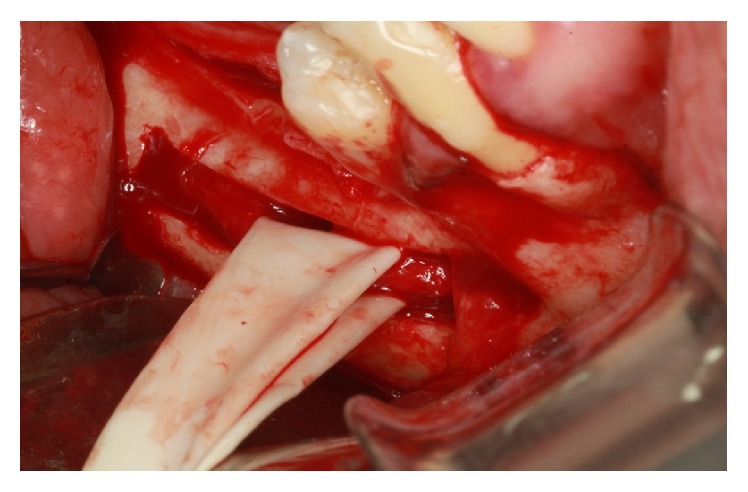
Traction beam performed with latex.

The cleavage of the cortical bone was performed with a Freer. With a Freer and a sterile glove fragment, the beam was pulled buccally to the milling procedure and the implant placement (Figures 27 and 28).

**Figure 29:**
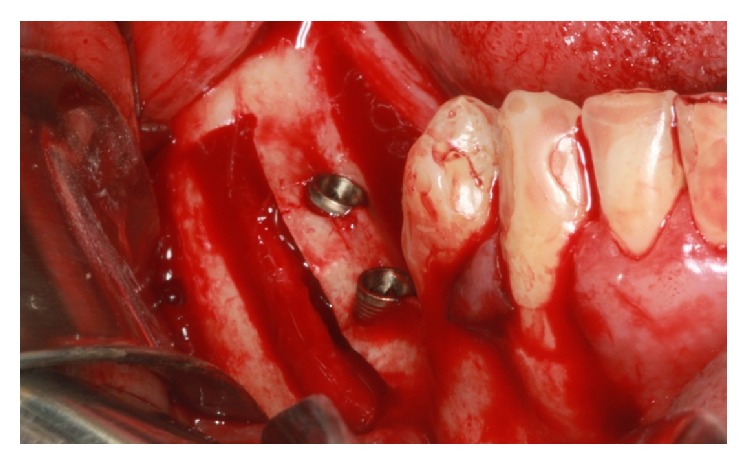
Inferior alveolar nerve positioned.

**Figure 30:**
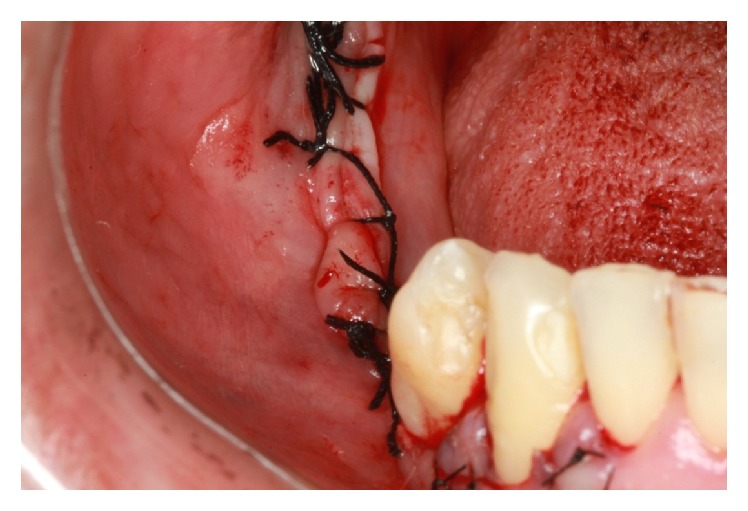
Suture.

The implants were installed in the regions of teeth 45 and 46 (Implant Torq®-Connection-3.5 mm × 13.0 mm). The IAN was repositioned on the implants (Figure 29). The flap was repositioned with a continuous scalloped suture using 3-0 silk thread (Ethicon) and 5-0 nylon thread (Ethicon) in the relaxing incisions (Figure 30).

#### 3.2.5. Postoperative Recovery

**Figure 31:**
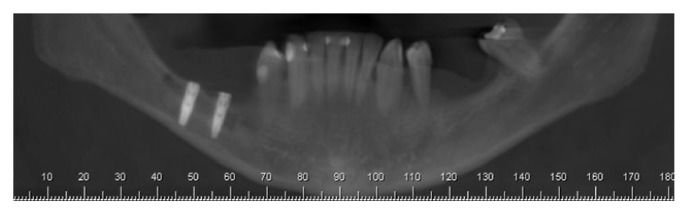
Panoramic court after implant placement.

**Figure 32:**
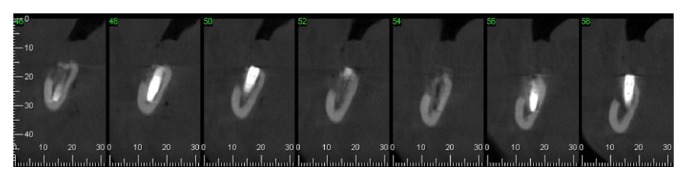
Computed tomography (cone beam) of the right mandible.

**Figure 33:**
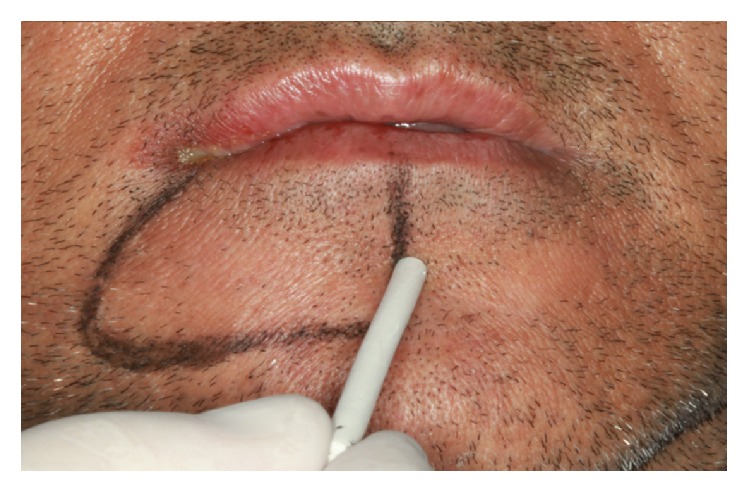
Tactile discrimination test.

**Figure 34:**
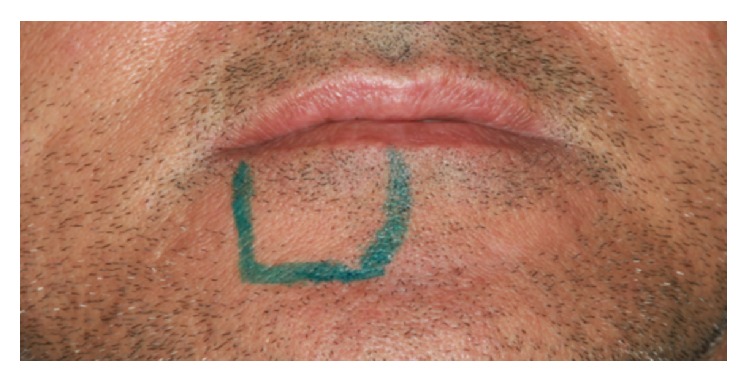
Demarcation of sensory change area in the 28-day postoperative period.

The patient was advised to make use of anti-inflammatory nimesulide (nimesulide, Medley, 100 mg), 1 tablet every 12 hours for 5 days, and analgesic dipyrone (sodium dipyrone, Medley, 500 mg), 30 drops every 6 hours for 2 days. After surgery, a CT scan was performed to evaluate the implant (Figures 31 and 32). Twenty four hours postoperatively, the patient had no pain—only a small, localized edema. In the evaluation of sensorineural disorder, a directional test with a brush and light touch pressure with a gutta percha cane were used (Figure 33). In the directional test, the patient was able to distinguish the direction of the vertical and horizontal movements. The light touch test served to demarcate the area corresponding to the sensory change. During examination in the 24-hour postoperative period, the patient suggested a hypoesthesia frame. At the 14-day follow-up, there was a decrease in the area corresponding to the sensory abnormalities, which occurred again at the 28-day follow-up. After three months, the patient had no sensory damage (Figure 34).

## 4. Discussion

The installation of dental implants is directly related to the amount and quality of bone present in the region to be restored. Later surgeries on extant edentulous jay are challenging due to the high degree of atrophy of the alveolar bone, preventing the installation of implants in the region. This is especially so in cases where the anatomical limitation has been caused by the presence of the mandibular canal and its contents, the IAN [11, 23, 24].

Some treatment options can be used for reconstruction of bone: guided bone regeneration, short implants, laterally tilted implants installed near the nerve, distraction osteogenesis, and IANT or LIAN [1, 14, 19, 25–30].

However, in the reconstruction with bone grafts, it is difficult to predict the gain of the alveolar crest due to difficulties in coating and bone quality [25]. Short implants have high failure rates for biomechanical problems as well as for bone quantity and quality [31]. The installation of the laterally inclined nerve implant is limited by abutments and it is at increased risk of biomechanical failure [23]. Distraction osteogenesis is a complex technique that requires great patient cooperation and two operations [32].

IANT and LIAN are techniques that most satisfy the later rehabilitation of atrophic jaws. In these procedures, the implant placement occurs in the correct position or as close as possible to the ideal, improving for a direct view at the time of surgery, unlike the implants inclined laterally to the nerve [17]. Using the upper and cortical basal body of the jaw, the implant is encased in a better-quality bone, unlike the implants installed in the reconstruction of regions with short grafts and implants. Furthermore, implants have better distribution of occlusal loads, favorable biomechanics, a high success rate, a single operative step, a shorter treatment time, a smaller cost, and less patient morbidity [18].

The disadvantages of the LIAN and IANT techniques are associated with potential complications such as sensorineural dysfunction (reported by all authors), mandibular fracture [26], and osteomyelitis [33]. Chrcanovic and Custódio [12] reported that the surgical technique does not recover the original anatomy of the jaw, leading to an impaired aesthetic of prosthetic rehabilitation.

According to the authors consulted [8, 30, 34, 35], the execution of the osteotomy with the piezoelectric device promotes a simpler and safer approach in the surgical techniques of LIAN and IANT compared to techniques using conventional rotary instruments, as they (in this case, the IAN) promote a bone cut without causing injury to the soft tissue. In cases of LIAN and IANT surgeries, the flaps required for IAN access to the cortical bone create a smaller exposure area. They also increase the possibility of making a smaller bone window, decrease the nerve traction in the chin, and decrease the possible sensorineural damage, all the while preserving a larger amount of the remaining bone, thereby preventing mandibular fracture.

For the analysis of the neurosensory function of IAN, the most commonly used test is two-point discrimination, as reported by several authors [13, 14, 17, 23, 24, 26, 29, 30, 33, 34]. Other objective tests were used such as the light touch test light, the heat test, the cold pin prick test, and the pressure test, as reported by several authors [8, 17, 26, 31, 33]. Nocini et al. [36]. Aside from these tests, a test was conducted to measure objective electrophysiological nerve conduction velocity and sensory action potential.

In addition to the objective tests, Kan et al. [14], Nocini et al. [36], Morrison et al. [17], Ferrigno et al. [26], and Hashemi [29] used a subjective analysis through a simple questionnaire, which patients used to report the presence or absence of pain, paresthesia, anesthesia, hypoesthesia, hyperesthesia, or dysesthesia. In this study, a sensorineural disorder patient was assessed by the light touch test to diagnose the type of nerve fibers damaged by the surgical procedure. The tactile discrimination test was also conducted to delimit the area affected by sensory damage in the case of hypoesthesia. Monitoring during the postoperative period was performed using the two-point discrimination test.

Some LIAN procedures showed no sensory damage in the postoperative period, while in other procedures sensitivity returned in a month. Ferrigno et al. [26] performed 19 LIAN procedures and, through subjective and objective testing (light touch, pain, and two-point discrimination), observed that, after anesthesia, no sensory damage had occurred in nine of the procedures. It occured after a month in two patients and in one procedure the patient reported sensory damage and permanent hypoesthesia.

In some IANT reports, all patients had sensory damage postoperatively. Without the use of any test, Friberg et al. [1] observed in 10 IANT procedures that all operated regions had a total loss of sensation one week postoperatively. A month later, two regions had completely returned to normal, and after six months, 70% of the regions had total sensory normality. Also without the use of any test, Chrcanovic and Custódio [12] presented the results of 18 IANT procedures using the conventional technique with drills. Patients underwent low-intensity laser application and Citoneurin© 5000 IU. All patients reported the initial change in sensitivity (paresthesia) and observed total recovery of sensitivity within six months (1 case in 2 months; 7 cases in 4 months; 5 cases in 5 months; and 5 cases in 6 months).

The success rate reported in the LIAN and IANT implant techniques ranged from 96% to 100%. However, in the IANT procedures, some authors observed lower success rates. The case reports and literature review showed that the LIAN was suggested to be much safer than IANT. Friberg et al. [1] reported a rate of 86.60%, Rosenquist [23] reported a rate of 93.60%, Hirsch and Brånemark [33] reported rates of 80% and 100% for IANT and LIAN, and Kan et al. [14] reported an average implant success rate of 93.89% and noted that a higher rate of implant loss occurred in IANT as compared to LIAN.

## 5. Conclusion

The inferior alveolar nerve transposition technique has a higher initial rate of sensorineural dysfunction than the lateralization technique for the inferior alveolar nerve, but in this case report, the two techniques showed similar sensory feedback. The authors found that the implant success rate is linked to the possibility of installing implants with long bicortical anchor, which favors primary stability and biomechanics.
